# Institutional Notes and News

**Published:** 1911-06-24

**Authors:** 


### Institutional Notes and News.

**A Wing "To Let" at Cardiff Infirmary.**

Colonel Bruce Vaughan, in speaking to a large audience last week at the annual meeting of the Cardiff Infirmary Needlework and Linen Guild, said that the new wing which had just been added to the infirmary needed an income of £7,000 per annum to keep it going, and out of this sum he was pleased to say they had secured £3,000. They only wanted another £4,000 a year to keep that great building going. The new wing could not be opened until the full income was forthcoming. He had suggested that on all the windows of the new wing notices should be displayed, "To Let." He added that there were now 230 men, 320 women, and 224 children waiting for admission to the infirmary, giving a total of 784 on the expectancy list.

**York Hospital and State Insurance.**

The action of the British Hospitals Association in calling the special meeting in London to consider the Government's Insurance Bill was referred to last week at the quarterly Court of the York County Hospital. The report of the House Committee, which was read by Mr. Neden, the secretary, whose contribution on the Insurance Bill appeared in these columns last week, contained the following: "It is difficult to say what the effect will be on the workpeople's contributions (whose present grant of £150 was acknowledged gratefully) if the National Insurance Bill passes into law in its present form? . . . The question is so important that at a meeting of the British Hospitals Association, recently held in London, a committee was appointed to arrange for a deputation to wait upon the Chancellor of the Exchequer to bring to his notice the damaging effect the Bill will have upon the finances of voluntary hospitals, unless some drastic alterations are introduced." The report further records the first official visit of the Archbishop of York to the hospital, which took place on May 31. His Grace, it states, made a tour of the wards. An additional chaplain, for Free Church patients, has been appointed. The Dean, the Very Rev. A. P. Purey-Cust, who presided, as usual, was congratulated on having reached his eighty-third birthday.

**Doings at a Yorkshire Fever Hospital.**

Thoughtful people of all shades of opinion are in one mind upon the point that it is false economy to endeavour to maintain and manage hospitals with small staffs and small expenses. Unfortunately there are many in public positions who, abject before the ratepayers, are persistent in their demands for curtailment. Amidst the rough hilly country of the Craven district of Yorkshire stands the little village of Austwick. There is situated a fever hospital which, at the moment of writing, is greatly troubling certain economists, for the reason that the establishment contains more nurses than patients. Some urgent appeals are being made for a reduction of staff, and a local journalist takes up the cudgels in defence of a maintenance of the present conditions. Whilst not pleading for the keeping up of an expensive and unnecessary establishment, he declares that economy can be carried too far, and that a large enough staff should be maintained to cope with possible emergencies. No one can tell when there might be an influx of patients which would necessitate even a larger staff than there is at present. But it appears that the minds of certain local councillors are disturbed not merely by this. The nurses have asked to be supplied with tennis equipment. A section of the Council has expressed its disapproval in the most decisive terms, and the local journalist finds an opportunity for supporting the nurses. The exacting conditions under which the nursing profession labours are such as to merit some due regard at the hands of those who govern the affairs of our public hospitals. Sometimes, indeed, we have had to remind the voluntary hospitals, as in the case of the St. Cross Hospital, Rugby, of similar facts.

**Hospital Fete at Wimbledon.**

Mr. and Mrs. W. A. Horn lent the gardens of their house at Wimbledon for the Coronation Fête which was held in aid of the building fund of the Cottage Hospital at Copse Hill. A new institution is replacing the old hospital, which want of room has made inadequate to its work. The fête, which lasted for two days, was opened on the first by H.R.H. Princess Louise, Duchess of Argyll, who, with the Duke, was received by Mr. J. F. Schwann, the chairman of the hospital, and of the fête committee, and by Councillor J. M. Bathgate, the Mayor. The Princess then received purses, the total contents of which amounted roughly to £300. One purse, presented by Miss D. Baig, contained £100. On the second afternoon the fête was opened by H.H. the Ranee of Sarawak.

**Jubilee of a Streatham Hospital.**

The treasurer of the British Home and Hospital for Incurables, at Streatham, Mr. F. A. Bevan, took the opportunity offered him by the recent jubilee dinner to explain the hopes of the institution. He recalled that there were now 377 persons each of whom was receiving £20 a year, and said that they would like greatly to add to their number. The income, he said, from subscriptions and donations now amounted to £10,000 a year, and of late years legacies had reached a similar sum. The income from investments was £3,400, and the cost of maintenance was £15,000 a year. Queen Alexandra, very soon after her first arrival in England, became its patron, and had been an annual subscriber. A telegram was despatched to her Majesty with the Home's congratulations. Mr. Justice and Lady Warrington were present.

**To-day's Ceremony at Charnwood.**

We are informed that another distinct step of progress has been made by the Leicester Saturday Hospital Committee, in the building of a new Convalescent Home for Women, the commemoration stones of which will be laid to-day (Saturday) by Sir Edward Wood, J.P., the president of the Society, with Mr. Orson Wright, J.P., and Mr. Wm. Newbery, the trustees appointed by the late Mr. Edward Higgs. The site chosen is at Woodhouse Eaves, Charnwood Forest, where the home will dominate the beautiful Swithland Woods. A home for women has long been needed by the Society, as may be imagined from the fact that over 400 women were sent to various convalescent homes by the Society last year. It will be of interest to recall that the late Mr. Higgs bequeathed to his trustees £10,000 to purchase a site and to build a convalescent home for consumptive patients at Cromer. The sum being found quite insufficient for the purpose, the trustees, with the consent of the Saturday Hospital Society approached the Court of Chancery and obtained sanction to vary the terms of the bequest with a view to the establishment of a Convalescent Home for Women in Leicestershire. The trustees were authorised to spend a portion of the capital sum and accrued interest in the purchase of a site and in the erection of a home. The balance was to be applied to its permanent maintenance. The Saturday Hospital Society purchased an additional adjoining site, and the home now being built stands on nine acres of ground. The cost of completion and furnishing is expected to be £17,000.

